# Seven Bacteriophages Isolated from the Female Urinary Microbiota

**DOI:** 10.1128/genomeA.01003-16

**Published:** 2016-11-23

**Authors:** Kema Malki, Emily Sible, Alexandria Cooper, Andrea Garretto, Katherine Bruder, Siobhan C. Watkins, Catherine Putonti

**Affiliations:** a Department of Biology, Loyola University Chicago, Chicago, Illinois, USA; b Bioinformatics Program, Loyola University Chicago, Chicago, Illinois, USA; c Department of Computer Science, Loyola University Chicago, Chicago, Illinois, USA

## Abstract

Recent research has debunked the myth that urine is sterile, having uncovered bacteria within the bladders of healthy individuals. However, the identity, diversity, and putative roles of bacteriophages in the bladder are unknown. We report the draft genome sequences of seven bacteriophages isolated from microbial communities from adult female bladders.

## GENOME ANNOUNCEMENT

Recently, we found seven phages from the bladders of four adult women with urge urinary incontinence (UUI) (1). Four independent bacterial cultures were grown in tryptic soy broth under anaerobic conditions at 37°C for 48 h. Supernatant was spotted (10 µl) on overlay plates of *Escherichia coli* C. Individual plaques were isolated and purified through successive plating on *E. coli* C. Six of the seven phages exhibited high proficiency in infecting and propagating within *E. coli* C. One phage (strain Wrath) was distinctly less fecund than the others on the *E. coli* host.

For each bacteriophage, liquid cultures of *E. coli* C were inoculated and grown overnight, with shaking, at 37°C; cultures were chloroformed, filtered (0.22 µm), concentrated via tangential flow filtration (0.10 µm), and treated with DNase I (Thermo, Fisher). DNA was extracted using the UltraClean Microbial DNA Isolation Kit (Mo Bio Laboratories) to produce the 1 ng of DNA used for library preparation (Nextera XT DNA Library Preparation Kit; Illumina), following the manufacturer’s instructions. Sequencing was performed on the Illumina MiSeq platform using the MiSeq reagent kit v2 producing 2 × 250 paired-end reads. Reads were trimmed using Geneious (Biomatters Ltd.) and assembled using SPAdes version 3.7.1 (2) with the careful option for *k* values ranging from 21 to 127. To confirm assemblies, trimmed reads were mapped to the *de novo* assembly via Bowtie2 (3). Genomes were annotated using RAST (4). No tRNA coding regions were detected (5).

The seven bacteriophage genomes include three distinct groups: Wrath (34.8% GC, 29,238 bp), Greed (44.6% GC, 60,042 bp), and the group including Sloth, Envy, Pride, Gluttony, and Lust (~54.5% GC, ranging in length from 41,942 to 45,206 bp). The genome sequence of Wrath was found via BLAST to most closely resemble the annotated *Bacillus cereus* D17 prophage (BLASTn query coverage of 79% and sequence identity of 82%), which includes the HK97 family major capsid protein. Homologous coding regions were also identified in the *Bacillus* phages BMPtp4 (KT372714) and BMPtpLA4 (KX190835). The presence of an integrase within the Wrath genome suggests it is able to exist as a temperate phage. This may indicate why even when grown in large volumes, consistently low coverage of the phage genome was retrieved (<100×). In contrast, high coverage was obtained for the other six phages, ranging from 120× (Greed) to 1507× (Sloth). Greed resembled one of the viruses (slur01) isolated from cattle slurry (6), relatives of coliphages Seurat (7) and CAjan (8). Sloth, Envy, Pride, Gluttony, and Lust also closely resembled a virus isolated from cattle slurry (6), slur05. Transmission electron microscopy (TEM) of the seven phages demonstrated that these viruses are tailed phages (*Caudovirales*), most likely *Siphoviridae*, as previously determined for Seurat (7), CAjan (8), and the *Bacillus* phages.

The bacteriophages presented here begin to describe the viral fraction of the complex communities within the human bladder.

### Accession number(s).

This whole-genome shotgun project has been deposited in DDBJ/ENA/GenBank under the accession numbers KX534335 (Envy), KX534336 (Gluttony), KX534337 (Greed), KX534338 (Lust), KX534341 (Pride), KX534339 (Sloth), and KX534340 (Wrath).
